# Supplementary figures and images for: Full atomistic model of prion structure and conversion
Source: PLoS Pathog. 2019 Jul 11;15(7):e1007864. doi: 10.1371/journal.ppat.1007864 (PMC6622554; doi:10.1371/journal.ppat.1007864)

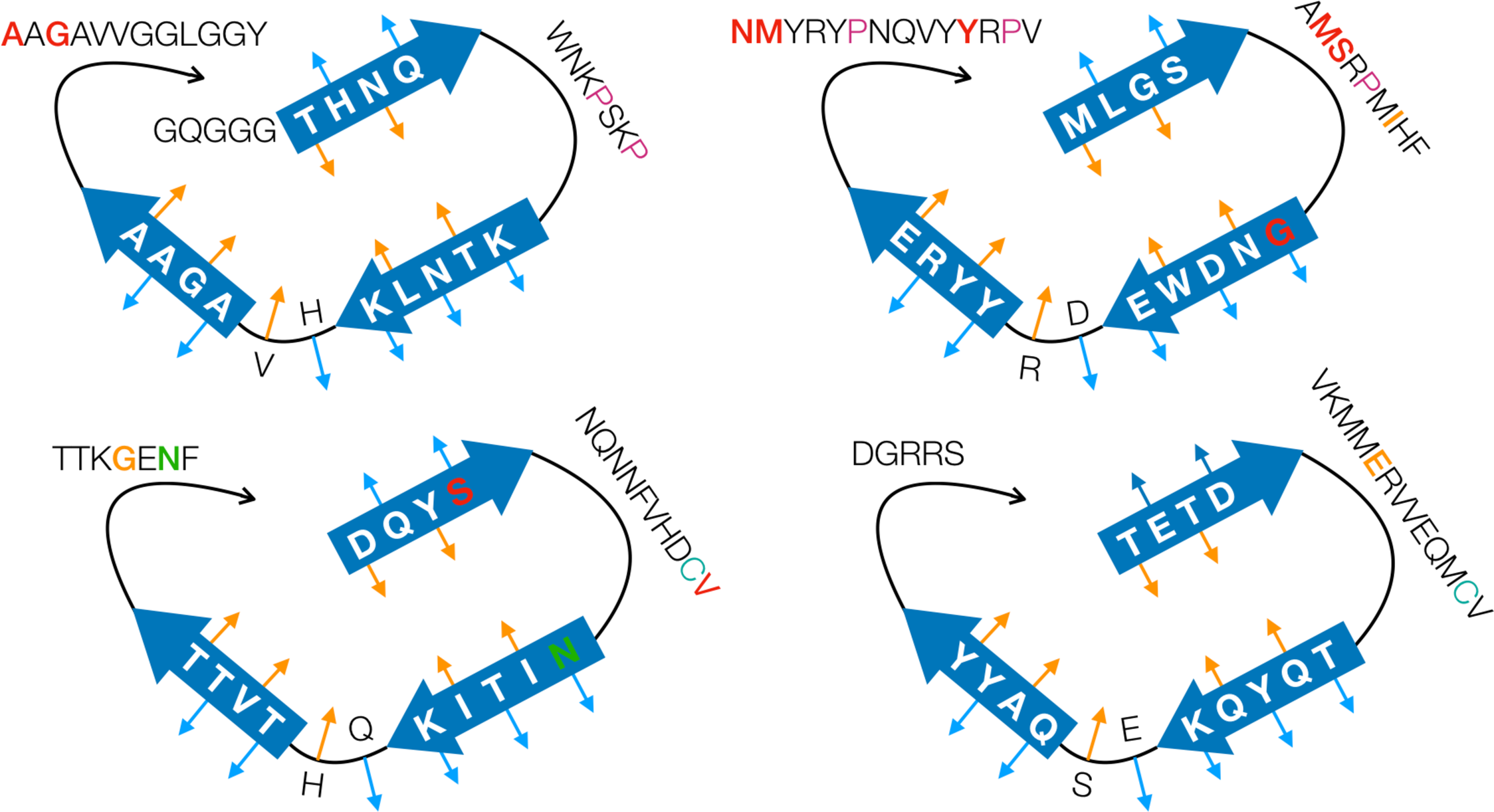

Supplement: S1 Fig — 2D arrangement based on the general architecture of right-handed β-solenoid proteins with L-shaped cross section. Blue thick arrows indicate β-strands, thin cyan and thin orange arrows indicate the sidechain orientation (toward the solvent or toward the hydrophobic core, respectively). The scheme is used to thread the moPrP 89–230 sequence by considering different constraints. PK cleavage sites identified by mass spectrometry in two different previous reports are colored in red. Glycosylation sites are labelled in green. Prolines are colored in purple. Cystine is indicated in cyan. (TIF) [file ppat.1007864.s001.tif]

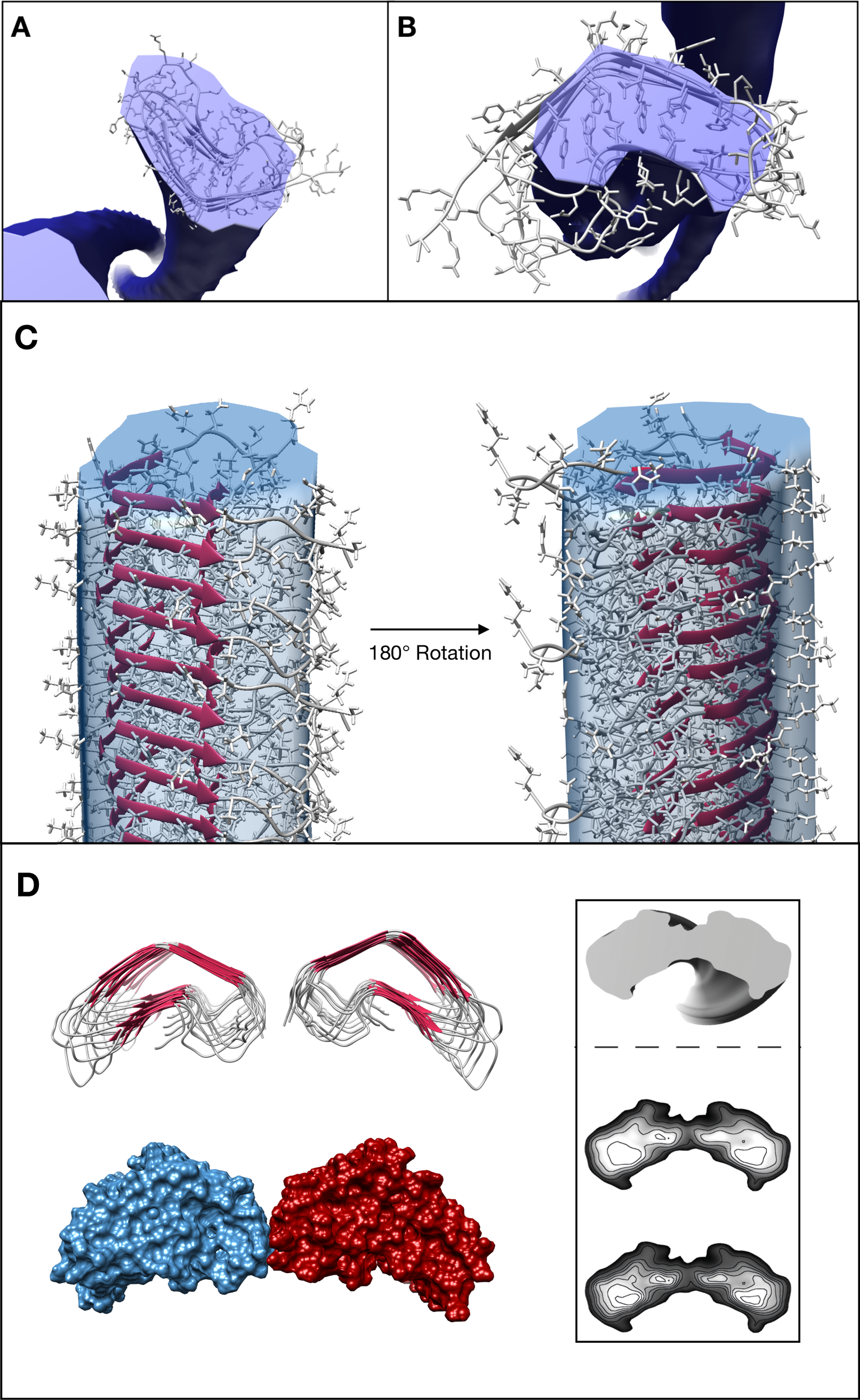

Supplement: S2 Fig — The pictures illustrate that the size of 4RβS fits with the protofilament cross section (50 Å x 30 Å) obtained by cryo-EM. (A) Level of contouring was set to 3.05 to match the measured protofilament diameter as determined from the raw electron micrograph [4]. (B) Level of contouring was tightened to 3.60 to highlight the superimposition of the hydrophobic core of the 4RβS with the core electron density region of the protofilament. (C) Reconstruction of a PrPSc protofilament superimposed to the cryo-EM map. (D) Cross-sectional view of two PrPSc protofilaments compared with the orientation reported in [4]. (TIF) [file ppat.1007864.s002.tif]

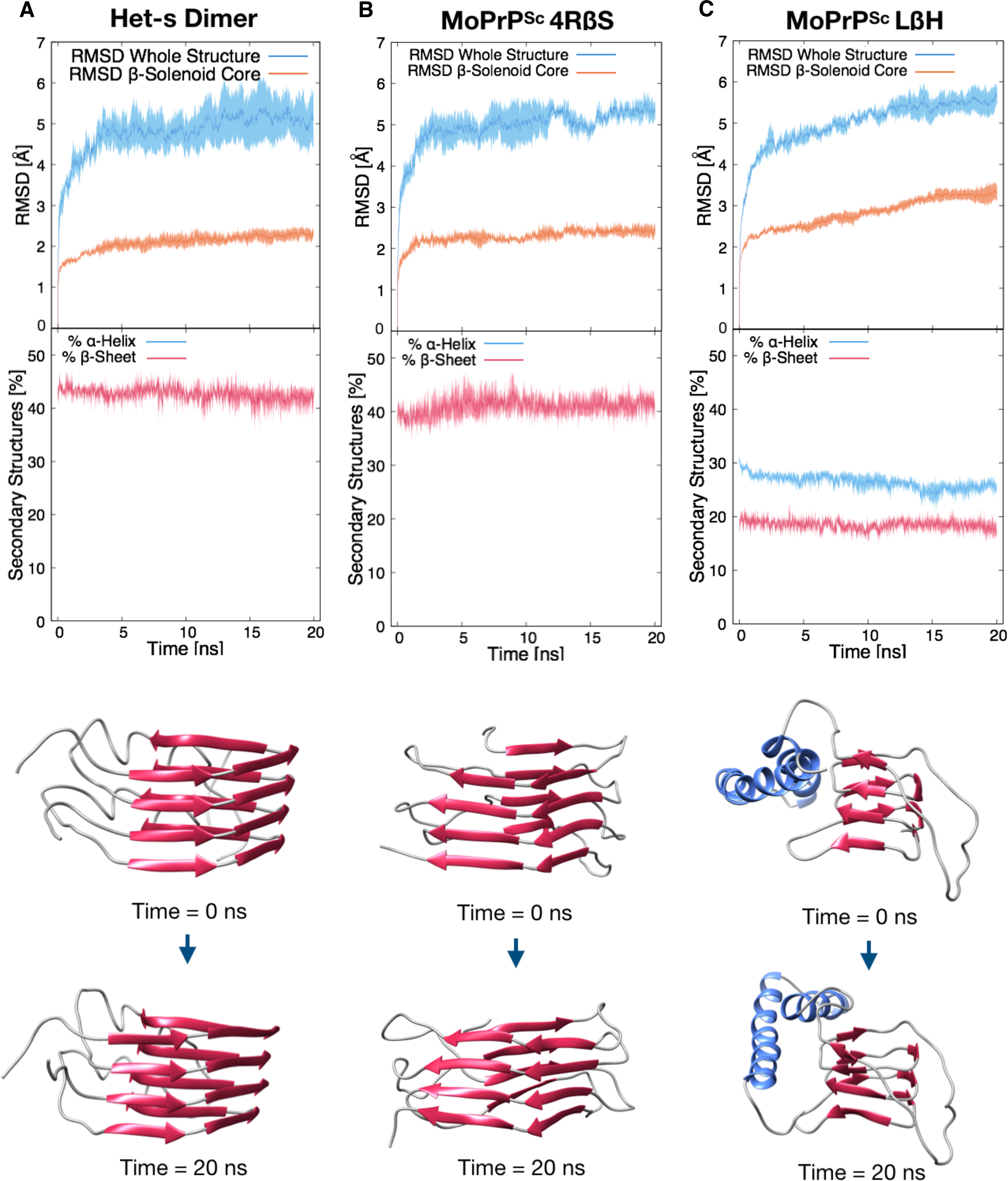

Supplement: S3 Fig — Upper graphs report the RMSD deviation from initial configurations for the entire structure (blue lines) or the β-solenoid core (orange lines) of the different proteins during the 20 ns of restrained MD simulations. Filled curves represents standard error of the mean. The graphs indicate a minor rearrangement of the β-solenoid core that is almost identical for the Het-s dimer and the 4RβS model, characterized by a RMSD of 2.3 ± 0.2 Å and 2.4 ± 0.2 Å respectively (calculated as the average of the three trajectories over the last 5 ns, ± standard deviation). In contrast, the hydrophobic core of the LβH model displays a higher RMSD deviation (3.3 ± 0.3 Å). Lower graphs indicate the α-helical (blue lines) or β-sheet (red lines) content of each protein. The latter is stable for all the three structures, likely due to the presence of the restraining potential for the whole length of the three simulations. (TIF) [file ppat.1007864.s003.tif]

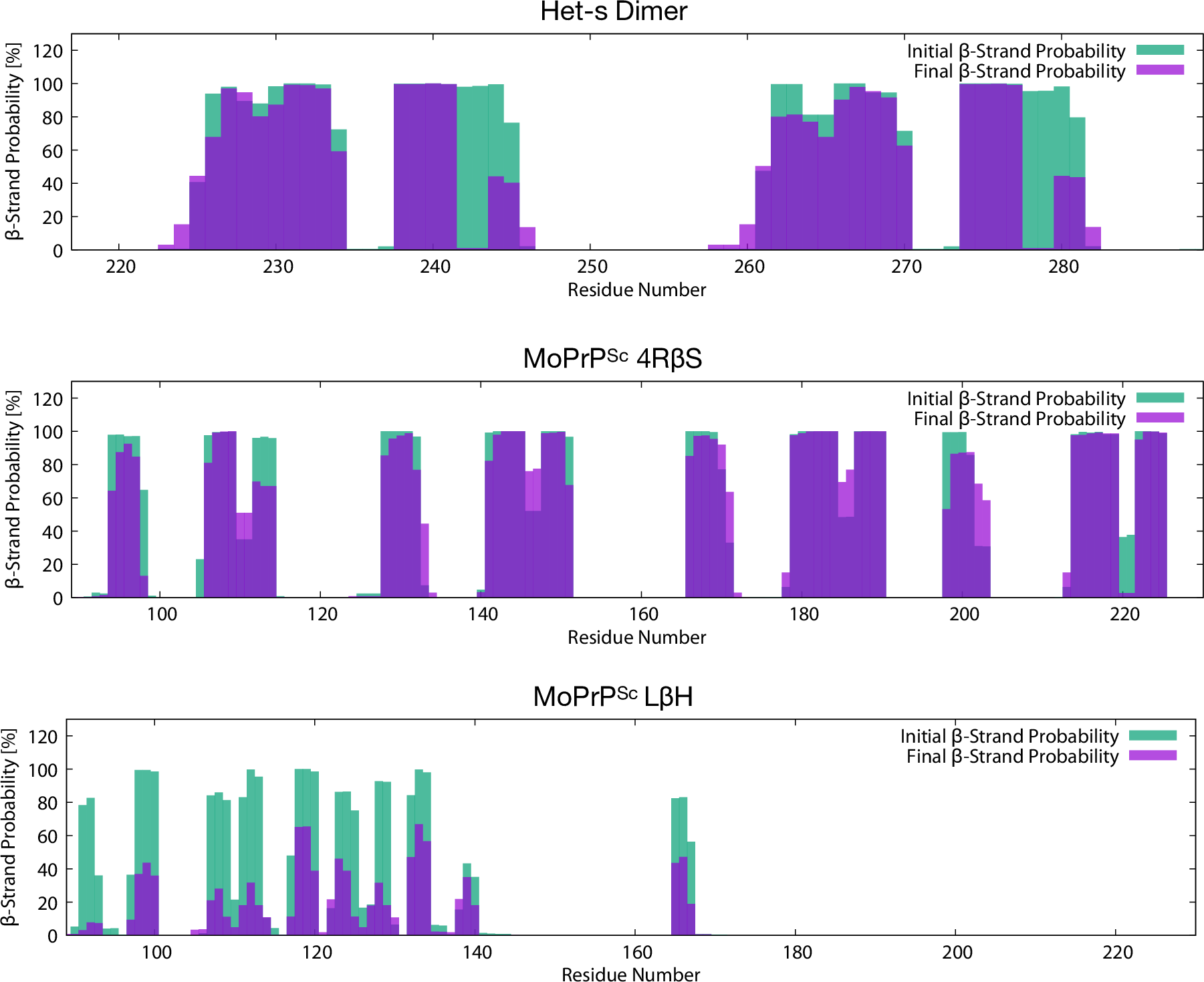

Supplement: S4 Fig — Graphs show the probability of each residue to be in an extended conformation at the beginning of the simulation (green) or at the end of the simulation (purple) for the Het-s dimer, 4RβS and LβH PrPSc models. Initial or final probabilities were calculated from the last 5 ns of the restrained or unrestrained simulations, respectively. (TIF) [file ppat.1007864.s004.tif]

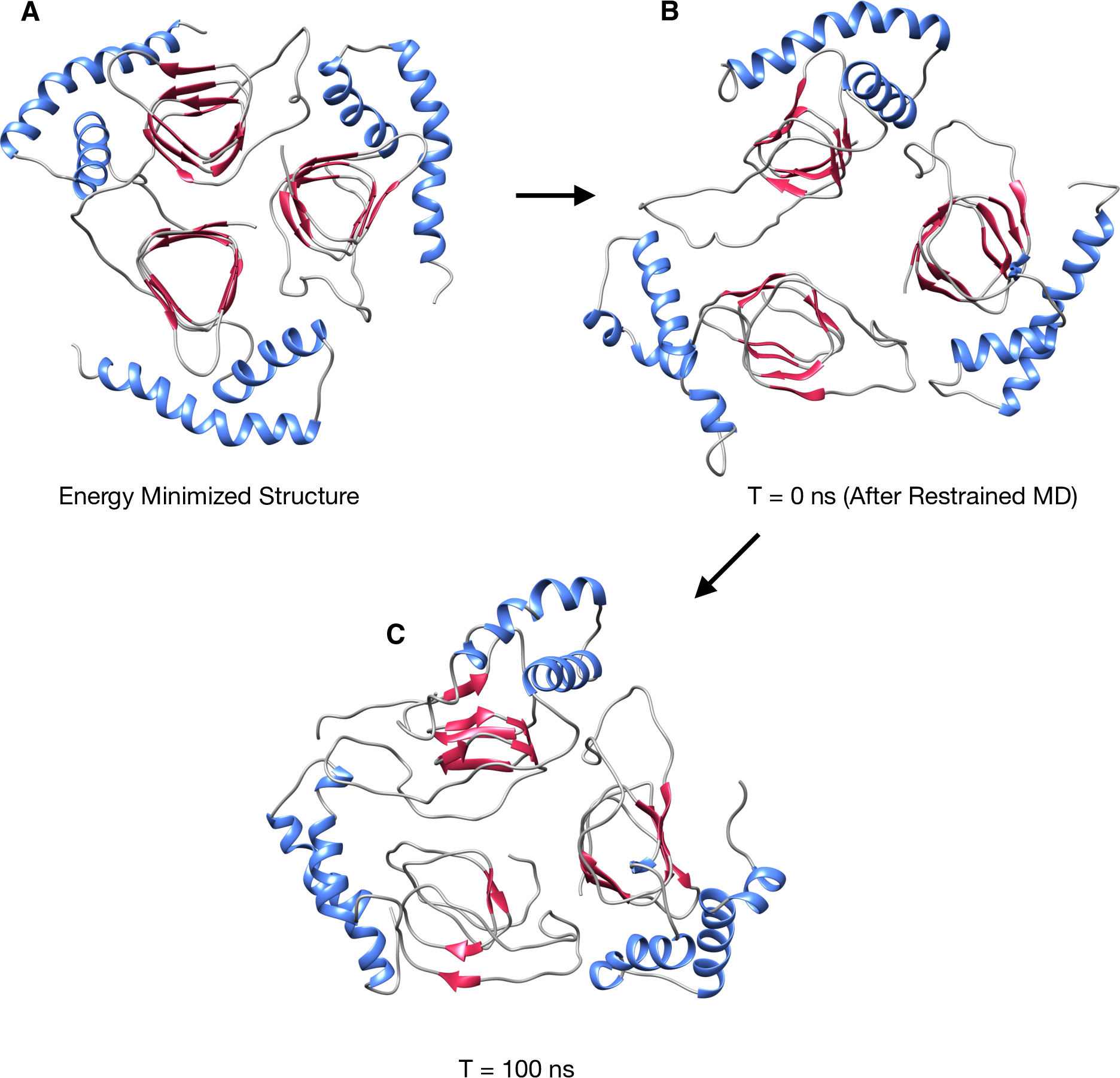

Supplement: S5 Fig — Pictures show the energy minimized structure (1), the same structure at end of the restrained (2) or unrestrained (3) MD simulations of the LβH model. (TIF) [file ppat.1007864.s005.tif]

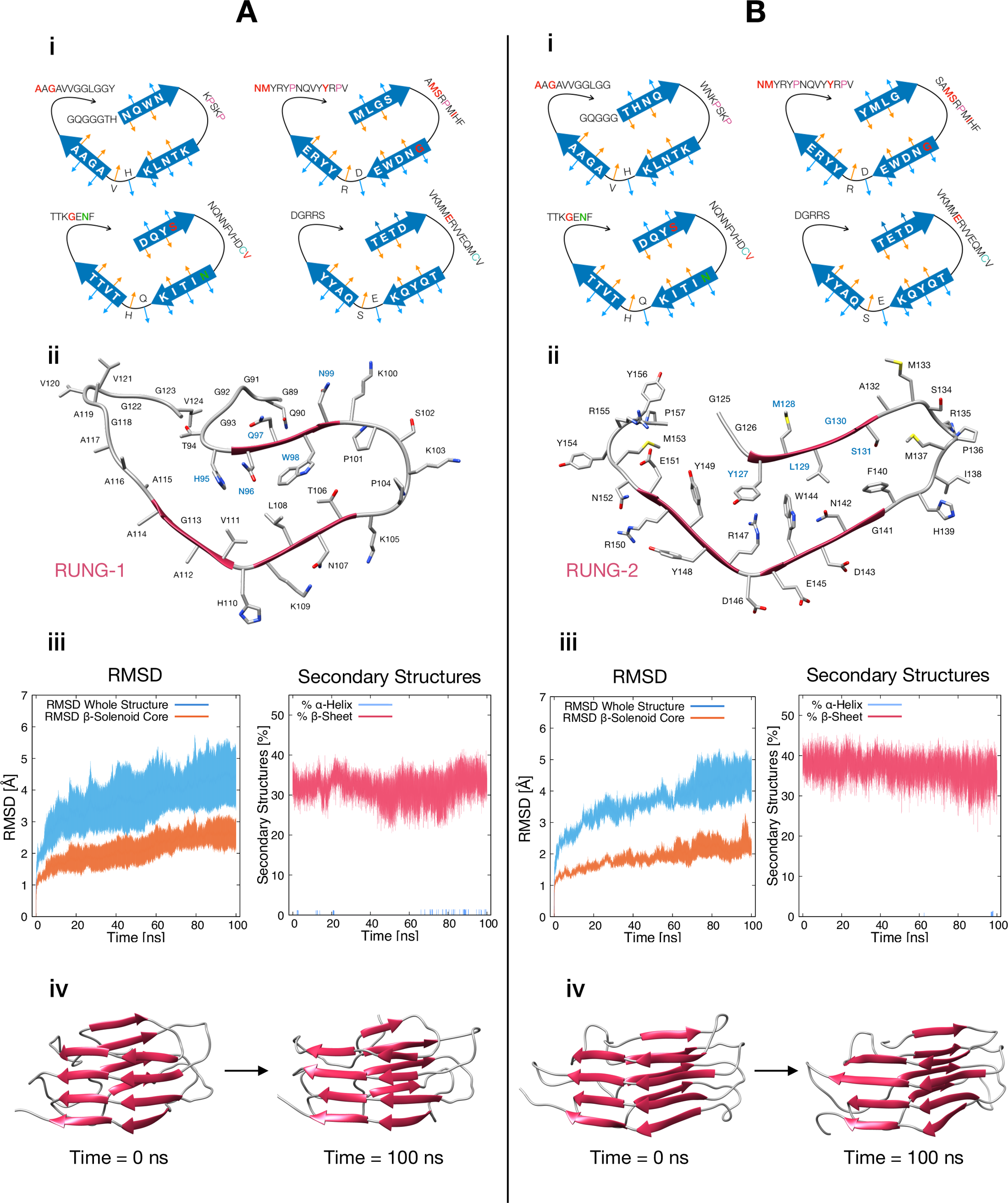

Supplement: S6 Fig — Panels A and B represent two possible alternative threading schemes not violating available experimental constraints. Only small deviations from the main model are allowed to agree with experimental data. Images (i) represents the 2D schemes of two putative alternative schemes. Images (ii) highlight the structure of the rung that differs from the main treading scheme, blue residues represent the shifted amino acids in the threading. Graphs (iii) report RMSD and secondary structure analysis of three 100 ns unrestrained MD simulations. Images (iv) report the initial and final frame of the respective MD simulations. (TIF) [file ppat.1007864.s006.tif]

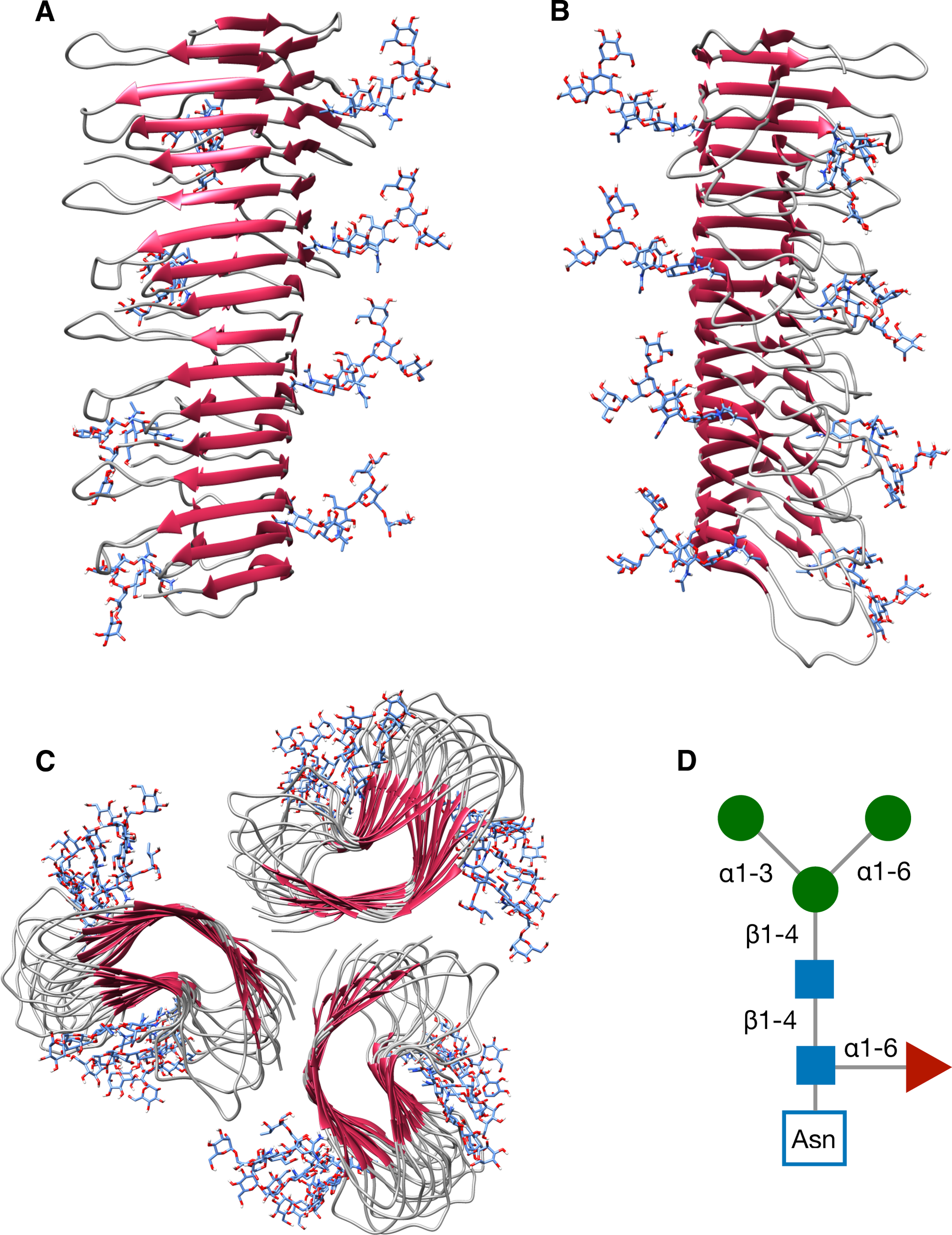

Supplement: S7 Fig — A model of energy minimized tetrameric 4RβS structure carrying glycan residues is depicted in two different orientations (A, B). Top view of a laterally stacked trimer of glycosylated 4RβS (C), compatible with the 2D crystals diffraction data. A scheme of the complex glycan precursor added is depicted in (D), blue squares indicate N-acetylglucosamine, green circles indicate mannose and the red triangle indicate fucose. (TIF) [file ppat.1007864.s007.tif]

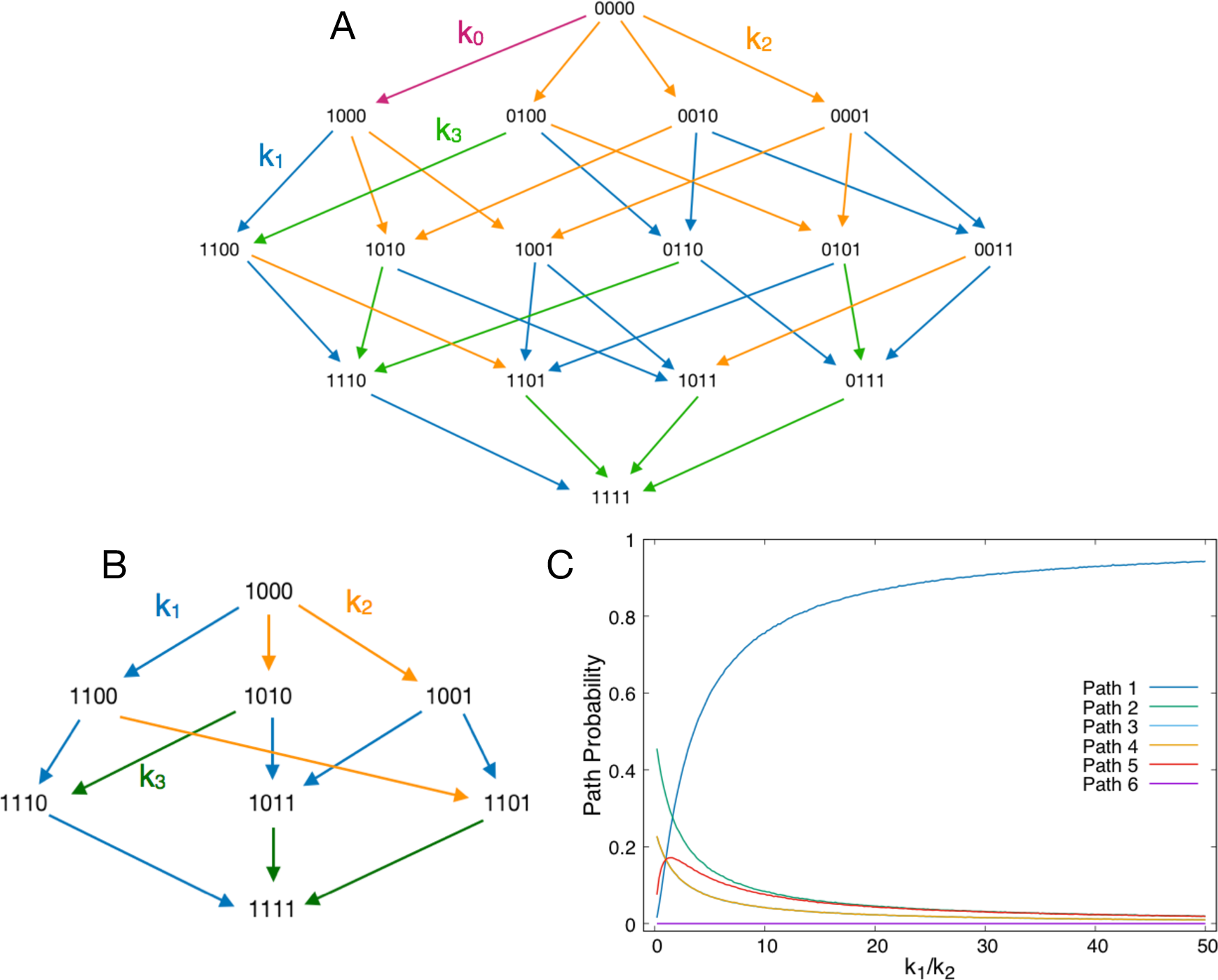

Supplement: S8 Fig — (A) Schematic representation of the network of transitions leading to the incorporation of PrPC into PrPSc. The purple arrow describes the formation of a single rung by templating the unstructured region of PrPC (89–115) on PrPSc (rate k0). The blue arrows describe the formation of a single by means of a templating process which involves the breaking the native contacts and the docking onto a pre-formed rung (rate k1). The orange arrows indicate the spontaneous formation and docking of two de-novo rungs (rate k2). Finally, the green arrows indicate the docking of preformed rungs belonging to two adjacent misfolded regions of the same chain (rate k3). (B) Simplified version of the network assuming as a priming reaction step the formation of the first rung. (C) Relative probability of the 6 reaction pathways as a function of the k1/k2 rate ratio. (TIF) [file ppat.1007864.s008.tif]
